# Circulating Interleukin-6 as a Prognostic Biomarker for Mortality in Melioidosis: A Systematic Review and Meta-Analysis

**DOI:** 10.3390/diseases13120385

**Published:** 2025-11-27

**Authors:** Supphachoke Khemla, Chaimongkhon Chanthot, Anchalee Chittamma, Jongkonnee Thanasai, Atthaphong Phongphithakchai, Moragot Chatatikun, Jitbanjong Tangpong, Sa-ngob Laklaeng, Wiyada Kwanhian Klangbud

**Affiliations:** 1 Division of Infectious Diseases, Department of Internal Medicine, Nakhon Phanom Hospital, Nakhon Phanom 48000, Thailand; sup.mednkp@gmail.com; 2 Project for the Establishment of the Faculty of Medicine, Nakhon Phanom University, Nakhon Phanom 48000, Thailand; chaimongkhon251269@gmail.com; 3 Department of Pathology, Faculty of Medicine Ramathibodi Hospital, Mahidol University, Bangkok 10400, Thailand; anchalee.chi@mahidol.ac.th; 4 Faculty of Medicine, Mahasarakham University, Mahasarakham 44000, Thailand; jongkonnee@msu.ac.th; 5 Nephrology Unit, Division of Internal Medicine, Faculty of Medicine, Prince of Songkla University, Songkhla 90110, Thailand; atthaphong.p@psu.ac.th; 6 School of Allied Health Sciences, Walailak University, Nakhon Si Thammarat 80160, Thailand; moragot.ch@wu.ac.th (M.C.); rjitbanj@wu.ac.th (J.T.); sangob.ll@wu.ac.th (S.-n.L.); 7 Research Excellence Center for Innovation and Health Products (RECIHP), Walailak University, Nakhon Si Thammarat 80160, Thailand; 8 Medical Technology Program, Faculty of Science, Nakhon Phanom University, Nakhon Phanom 48000, Thailand

**Keywords:** melioidosis, *Burkholderia pseudomallei*, interleukin-6, cytokine, sepsis, biomarker, mortality, systematic review, meta-analysis

## Abstract

**Background:** Melioidosis, caused by *Burkholderia pseudomallei*, remains a major cause of sepsis-related mortality in tropical regions. Despite effective antimicrobial therapy, deaths frequently result from dysregulated host inflammation rather than uncontrolled bacterial replication. Interleukin-6 (IL-6), a key mediator of systemic inflammation, has been proposed as a prognostic biomarker in sepsis, but its predictive value in melioidosis has not been systematically evaluated. **Methods:** A systematic review and meta-analysis were performed following PRISMA 2020 guidelines (PROSPERO CRD420251152797). MEDLINE, Embase, and Scopus were searched from inception to 15 March 2025. Eligible studies included patients with culture-confirmed melioidosis reporting circulating IL-6 concentrations stratified by survival outcome. Standardized mean differences (SMDs) with 95% confidence intervals (CIs) were pooled using random effects models. Heterogeneity and robustness were examined through leave-one-out and sensitivity analyses. Publication bias assessment was not performed due to insufficient study numbers *(n* = 4). **Results:** Eight studies were included qualitative systematic review, and four studies comprising 411 patients were eligible for quantitative meta-analysis. Pooled analysis demonstrated significantly higher IL-6 levels among non-survivors compared with survivors (SMD = 0.80, 95% CI 0.02–1.57; *I*^2^ = 86.3%). Leave-one-out diagnostics indicated no single study unduly influenced the pooled effect. Sensitivity analysis excluding the largest dataset reduced heterogeneity to 34.5% and yielded an SMD of 0.51 (95% CI −0.28–1.30), maintaining the same direction of association. **Conclusions:** Circulating IL-6 levels are elevated in fatal melioidosis and may serve as a promising prognostic biomarker for mortality. Although interstudy heterogeneity was substantial, the association remained consistent in direction across populations and analytical methods despite the limited number of eligible studies. These findings support further prospective validation of IL-6 in clinical risk stratification and host response-guided management of severe melioidosis, though larger multicenter studies are needed to confirm these preliminary findings.

## 1. Introduction

Melioidosis, caused by the Gram-negative bacterium *Burkholderia pseudomallei*, is a severe infectious disease that poses a major but under-recognized global health threat. Endemic to tropical soils and waters, particularly in Southeast Asia and Northern Australia, the geographic range of melioidosis is expanding as new endemic areas are identified worldwide [1,2]. Its clinical spectrum is remarkably diverse, ranging from localized abscesses to life-threatening sepsis with multi-organ failure, earning it the name “the great mimicker.” Despite availability of antimicrobial therapy, mortality in hyperendemic regions remains unacceptably high, often exceeding 40% in severe cases [1]. This high fatality rate suggests that death is not solely due to bacterial burden but also to dysregulated host immune responses. Experimental evidence demonstrates that *B. pseudomallei* actively manipulates host immunity through multiple mechanisms, including suppression of pyroptosis, inhibition of type I interferon responses, and interference with inflammasome activation [3,4]. These immune evasion strategies result in disproportionate leukocyte recruitment without effective bacterial clearance, contributing to immunopathology and organ damage [5].

The pathogenesis of severe melioidosis reflects the interplay between virulence factors and host immunity. *B. pseudomallei* employs sophisticated mechanisms—including Type III and Type VI secretion systems, actin-based motility, and formation of multinucleated giant cells—to survive intracellularly and spread between host cells [6]. On the host side, severe cases are characterized by hyperinflammatory responses, or “cytokine storm,” which can lead to endothelial damage, coagulopathy, and multi-organ failure—the principal drivers of death in sepsis [7]. More recent immunological studies confirm that dysfunctional cellular immune responses are associated with poor outcomes in acute melioidosis, while survivors exhibit more robust IL-17–mediated T-cell responses [8].

Although these studies have substantially advanced our understanding of melioidosis immunopathology, a critical gap remains regarding the prognostic utility of specific inflammatory mediators in predicting mortality. While the biology of *B. pseudomallei* virulence is well characterized [6], quantitative thresholds for host inflammatory biomarkers that reliably stratify mortality risk in melioidosis have not been established.

Among candidate biomarkers, interleukin-6 (IL-6) represents a particularly promising target for several reasons. First, IL-6 is a proximal mediator of the acute inflammatory response, directly released by macrophages and monocytes upon pathogen recognition, making it mechanistically relevant to melioidosis pathogenesis [7]. Second, IL-6 has demonstrated prognostic value across multiple sepsis contexts, including community-acquired pneumonia, Gram-negative bacteremia, and polymicrobial sepsis [9]. Third, preliminary evidence from individual melioidosis cohorts suggests IL-6 elevation in fatal cases, but these findings remain fragmented across diverse populations, with wide variation in reported concentrations, assay methodologies, and statistical approaches [10,11,12,13]. No systematic synthesis has quantified the overall strength and consistency of this association.

While IL-6 is recognized as a prognostic marker in sepsis broadly, several features make it particularly relevant to melioidosis pathophysiology. *B. pseudomallei* is a facultative intracellular pathogen that survives within macrophages and induces formation of multinucleated giant cells, triggering robust IL-6 production from infected mononuclear phagocytes [3]. Unlike extracellular pathogens where IL-6 primarily reflects LPS-induced acute phase responses, melioidosis-associated IL-6 reflects chronic intracellular infection and granuloma formation. Experimental evidence demonstrates that *B. pseudomallei* lipopolysaccharide and Type III secretion system effectors directly stimulate IL-6 gene expression in human monocytes at levels exceeding those induced by *E. coli* LPS [14,15]. Furthermore, melioidosis exhibits unique immunological features including necrotizing granulomas, abscess formation in multiple organs, and a propensity for relapse—all processes mediated by sustained IL-6 signaling [5]. In murine models, IL-6 mRNA expression correlates directly with bacterial burden and disease severity, with persistent elevation predicting mortality [14]. The high prevalence of diabetes mellitus in melioidosis patients (40–60%) adds another dimension, as diabetic individuals exhibit exaggerated IL-6 responses to *B. pseudomallei* infection with impaired resolution [16]. Thus, IL-6 in melioidosis reflects not only systemic inflammation but also specific pathogen–host interactions including intracellular bacterial persistence, granulomatous inflammation, and metabolic dysregulation.

To address this gap, we conducted the first systematic review and meta-analysis to quantitatively assess the association between circulating IL-6 concentrations and mortality in culture-confirmed melioidosis. By pooling data across diverse patient populations and study settings, we aimed to (1) provide a pooled estimate of IL-6’s prognostic effect size, (2) evaluate sources of heterogeneity across studies, and (3) clarify IL-6’s potential utility as a biomarker for risk stratification and clinical management in melioidosis.

This manuscript is organized as follows: Section 2 describes our systematic review methodology, including search strategy, eligibility criteria, data extraction procedures, quality assessment, and statistical analysis approach. Section 3 presents results of the study selection process, characteristics of included studies, quality assessment findings, and pooled meta-analytic estimates with sensitivity analyses. Section 4 discusses the clinical and biological interpretation of findings, contextualizes results within existing literature, examines sources of heterogeneity, and acknowledges limitations. Section 5 provides conclusions and outlines future research directions for translating IL-6 biomarker evidence into clinical practice.

## 2. Materials and Methods

### 2.1. Overview of Research Design and Registration

This study employed a systematic review and meta-analysis methodology following the Preferred Reporting Items for Systematic Reviews and Meta-Analyses (PRISMA) 2020 statement [17]. This methodological framework was selected as the most appropriate approach to synthesize existing evidence and derive robust, pooled estimates of the association between circulating interleukin-6 (IL-6) concentrations and mortality in patients with culture-confirmed melioidosis. The systematic review design enables comprehensive identification and critical appraisal of all relevant studies, while meta-analysis provides quantitative synthesis to overcome the limitations of individual studies with varying sample sizes and effect estimates.

This systematic review and meta-analysis was prospectively registered with PROSPERO (registration number: CRD420251152797) to ensure transparency of methods and reduce the risk of selective reporting. All reporting follows the PRISMA 2020 guidelines, with a completed PRISMA checklist provided to ensure comprehensive and transparent reporting of methods and results.

### 2.2. Search Strategy and Data Sources

A comprehensive literature search was conducted across multiple electronic databases from inception to 15 March 2025, including MEDLINE (via PubMed), Embase, and Scopus. The search strategy combined medical subject headings (MeSH) and free-text terms related to melioidosis, *Burkholderia pseudomallei* and interleukin-6. The primary search terms included “Melioidosis” OR “*Burkholderia pseudomallei*” combined with “Interleukin-6” OR “IL-6” OR “interleukin 6”. The search strategy was adapted for each database to optimize sensitivity while maintaining specificity, with no language or publication date restrictions applied to maximize comprehensiveness. The search strategies were in Supplementary File S1.

Beyond the primary database search, reference lists of included studies and relevant review articles were manually screened to identify additional eligible studies that may have been missed in the electronic search. The gray literature was systematically searched through conference lists. Expert consultation and communication with researchers in the field of melioidosis were also undertaken to identify unpublished studies or ongoing research that might contribute relevant data to the analysis.

### 2.3. Study Selection and Eligibility Criteria

Studies were systematically evaluated for inclusion based on predefined criteria addressing population, exposure, outcome, and study design characteristics. The inclusion criteria required studies to focus on patients with culture-confirmed melioidosis, defined as positive culture of *B. pseudomallei* from any clinical specimen, ensuring diagnostic certainty and reducing heterogeneity from misclassified cases. Studies must have measured circulating IL-6 concentrations in serum or plasma specimens at hospital admission or within 24 h of presentation and reported mortality data with clear definitions of survival status. Studies reporting only serial measurements at later time points (e.g., day 3, 7, 14) without baseline admission values were excluded to ensure temporal comparability. For studies with both admission and follow-up measurements, only admission/day 0 values were extracted for meta-analysis. Eligible study designs included observational studies such as cohort, case–control, and cross-sectional studies, as well as randomized controlled trials that reported IL-6 levels stratified by mortality outcome. No language restrictions were applied, and both published peer-reviewed articles and conference abstracts were considered eligible to minimize publication bias.

Studies were excluded if they represented case reports, case series, reviews, editorials, or commentaries that did not provide primary data. Additionally, studies lacking culture confirmation of *B. pseudomallei* infection were excluded to ensure diagnostic accuracy, as were studies without quantitative IL-6 measurements or adequate mortality outcome data. Studies with insufficient data for meta-analysis were excluded only after unsuccessful attempts to contact authors for additional information. In cases of duplicate publications from the same study population, the most comprehensive report was retained to avoid double-counting of participants.

Study selection was performed independently by two reviewers (W.K.K. and S.K.) at both title/abstract screening and full-text review stages. Inter-reviewer agreement was assessed using Cohen’s kappa statistic. At the title/abstract screening stage, agreement was substantial (κ = 0.82, 95% CI 0.74–0.90), with 156 of 170 records (91.8%) receiving concordant include/exclude decisions. At the full-text review stage, agreement was near-perfect (κ = 0.91, 95% CI 0.83–0.99), with disagreement on only 3 of 88 articles. All disagreements were resolved through structured discussion, with a third reviewer (A.C.) consulted for 2 articles where consensus could not be reached.

### 2.4. Data Extraction and Management

#### 2.4.1. Systematic Data Collection Process

Data extraction was performed independently by two trained reviewers using a standardized, pilot-tested extraction form developed specifically for this review. Prior to full data extraction, the form was piloted on a subset of studies to ensure consistency and completeness of data capture. Disagreements between reviewers were resolved through structured discussion, and when consensus could not be reached, a third reviewer was consulted for final adjudication. The extraction process was designed to capture all relevant information necessary for both qualitative synthesis and quantitative meta-analysis.

#### 2.4.2. Study and Patient Characteristics

Comprehensive study characteristics were systematically extracted, including first author, publication year, country of origin, and study design classification. Additional details regarding study period, clinical setting (hospital-based versus community-based), and specific patient enrollment criteria were recorded to understand the context and generalizability of findings. Patient demographic information including sample size, mean age, sex distribution, and relevant comorbidities were extracted when available. Disease severity measures, clinical presentations, and treatment protocols were also documented to facilitate subgroup analyses and interpretation of heterogeneity.

#### 2.4.3. IL-6 Measurement and Outcome Data

Detailed information regarding IL-6 measurement methodology was systematically captured, including specimen type (serum or plasma), timing of collection relative to hospital admission or symptom onset, and specific assay methodology employed (ELISA, chemiluminescent immunoassay, or other platforms). Manufacturer details and assay specifications were recorded when available to assess potential sources of analytical variation. IL-6 concentrations in survivors versus non-survivors were extracted as reported, including measures of central tendency (mean ± standard deviation, median with interquartile range) and any reported cut-off values with associated diagnostic performance metrics.

Mortality outcome data were comprehensively extracted, including overall mortality rates, duration of follow-up, and specific definitions of mortality used in each study. Both crude and adjusted effect estimates (odds ratios) with corresponding confidence intervals were recorded when available. For multivariable analyses, the specific confounding variables adjusted for in the models were documented to assess the robustness of reported associations and to inform interpretation of pooled estimates.

#### 2.4.4. Identification and Management of Overlapping Patient Cohorts

To prevent double-counting of patients in meta-analysis, we systematically identified potentially overlapping cohorts using four criteria: (1) recruitment site (same hospital or clinical network), (2) recruitment period (overlapping dates), (3) author overlap (shared senior/corresponding authors suggesting same research group), and (4) sample size consistency (subset relationship evident from reported numbers).

For each study, we extracted: institution name, city, recruitment start and end dates, and author affiliations. Studies were flagged as potentially overlapping if they met ≥2 criteria. For flagged studies, we conducted detailed comparison of inclusion/exclusion criteria, baseline characteristics, and outcome definitions.

### 2.5. Quality Assessment

Study quality was independently assessed by two reviewers using the Newcastle Ottawa Scale (NOS) for observational studies, which provides a comprehensive framework for evaluating methodological rigor across three critical domains: selection of study groups (maximum 4 points), comparability of groups (maximum 2 points), and ascertainment of exposure and outcome (maximum 3 points). Studies achieving a score of 7 or higher were classified as high quality, those scoring 4–6 points as moderate quality, and studies with fewer than 4 points as low quality. This classification system allows for sensitivity analyses based on methodological rigor and provides transparency regarding the evidence base supporting pooled estimates.

### 2.6. Statistical Analysis

#### 2.6.1. Meta-Analytic Approach and Data Synthesis

Meta-analysis was performed using random effects models based on the DerSimonian–Laird method to account for anticipated heterogeneity across studies arising from differences in patient populations, clinical settings, assay methodologies, and other study characteristics. This approach provides more conservative estimates than fixed-effects models and is more appropriate when substantial between-study heterogeneity is expected. When studies reported IL-6 concentrations as continuous variables, standardized mean differences (SMDs) with 95% confidence intervals were calculated to enable comparison across studies using different units or assay platforms. For studies providing dichotomous databased on predetermined IL-6 thresholds, pooled odds ratios were computed using the Mantel-Haenszel method.

IL-6 concentrations were measured using different assay platforms across studies. These assays differ in sensitivity, dynamic range, and calibration standards, precluding direct comparison of absolute values. We addressed this heterogeneity through three approaches: (1) standardized mean differences (SMDs) as the primary effect measure, which are dimensionless and enable pooling across different units and scales; (2) meta-regression (not performed due to insufficient studies, but planned) to formally test whether assay type explained heterogeneity; and (3) narrative synthesis documenting assay-specific characteristics to maintain transparency regarding methodological variability.

#### 2.6.2. Heterogeneity Assessment and Exploration

Between-study heterogeneity was comprehensively assessed using multiple complementary approaches. Cochran’s Q test was employed to test for statistically significant heterogeneity, with statistical significance set at *p* < 0.10 given the low power of this test. The I^2^ statistic was calculated to quantify the proportion of total variation attributable to between-study heterogeneity, with values interpreted as low (0–40%), moderate (41–60%), substantial (61–80%), or considerable (81–100%) heterogeneity according to established guidelines. Additionally, tau-squared (τ^2^) was calculated as a measure of between-study variance, providing insight into the absolute magnitude of heterogeneity.

Given the anticipated heterogeneity in IL-6 measurement approaches, we employed several strategies to enable valid cross-study comparison. Standardized mean differences (SMDs) were selected as the primary effect measure because they are unit-free and allow pooling of studies using different assay platforms (ELISA, electrochemiluminescence, bioassay) and reporting different units (pg/mL vs. ng/mL). For studies reporting medians and interquartile ranges rather than means and standard deviations, we applied validated mathematical transformations to estimate means and standard deviations, documenting this approach to maintain transparency. Timing of IL-6 measurement was standardized by including only admission/baseline samples, excluding studies that measured IL-6 only at later time points. Random effects modeling was employed a priori to account for expected between-study variance in patient populations (disease severity, comorbidities), clinical settings (tertiary centers vs. district hospitals), and geographic factors (strain variation, treatment protocols).

#### 2.6.3. Sensitivity Analyses

Comprehensive sensitivity analyses were undertaken to assess the robustness of pooled estimates. Leave-one-out analysis was performed to evaluate the influence of individual studies on the overall effect estimate, identifying studies with disproportionate impact on the results. Additional sensitivity analyses included restriction to high-quality studies only, analysis limited to studies with more than 50 participants to reduce small-study effects, and comparison of results using fixed-effects versus random effects models to assess the impact of the chosen meta-analytic approach.

#### 2.6.4. Publication Bias Assessment

Publication bias, representing the tendency for studies with statistically significant or favorable results to be more likely to be published, was evaluated using multiple complementary methods. Visual inspection of funnel plots was performed to assess for asymmetry that might suggest small-study effects or publication bias. Egger’s regression test was employed to statistically test for small-study effects, with statistical significance set at *p* < 0.05. Begg’s rank correlation test provided an additional statistical assessment of publication bias based on the correlation between standardized effect estimates and their variances. When evidence of potential publication bias was detected, trim-and-fill analysis was performed to estimate the number and effect of potentially missing studies and to provide adjusted effect estimates accounting for this bias.

#### 2.6.5. Statistical Software and Reporting Standards

All statistical analyses were conducted using R statistical software (version 4.3.0) with specialized packages including meta for general meta-analysis functions, metafor for advanced meta-analytic techniques including meta-regression, and dmetar for additional diagnostic and visualization tools. Results are presented as forest plots displaying individual study estimates alongside pooled effects, with clear indication of confidence intervals and measures of heterogeneity. Statistical significance was set at α = 0.05 for all primary analyses, with the exception of heterogeneity testing where α = 0.10 was used given the lower power of these tests.

## 3. Results

### 3.1. Study Selection and Identification

**Figure 1:**
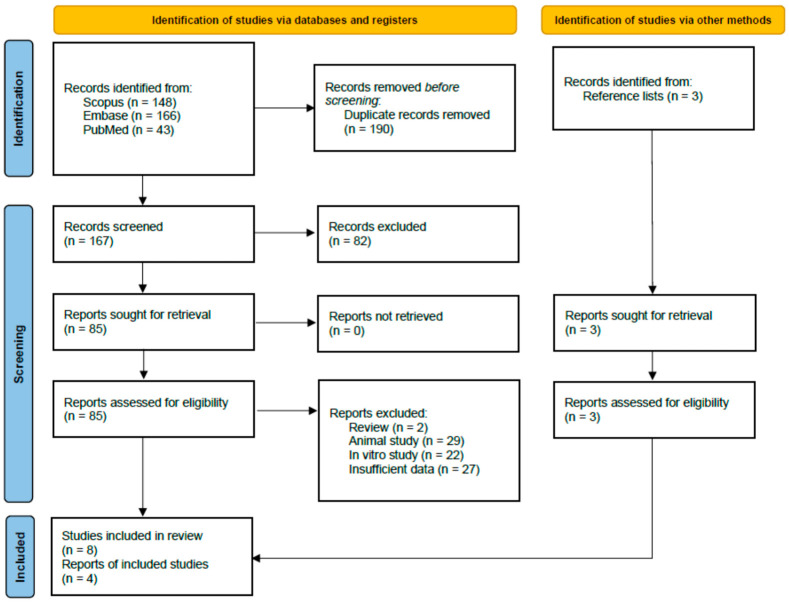
PRISMA 2020 flow diagram for study selection. The diagram details the process of identifying, screening, assessing for eligibility, and including studies in the systematic review and meta-analysis.

The systematic literature search identified a total of 360 records from PubMed (43), Embase (166), Scopus (148), and Supplementary Sources (3). After the removal of 190 duplicates, 170 unique records were screened based on title and abstract, leading to the exclusion of 82 records that were irrelevant to the research question. The full texts of the remaining 88 articles were retrieved and assessed for eligibility. Of these, 80 were excluded for various reasons: 2 were review articles without original data, 29 were animal studies, 22 were in vitro studies, and 27 were insufficient data for analysis. Eight studies met inclusion criteria for qualitative systematic review (Table 1), meaning they reported IL-6 measurements in culture-confirmed melioidosis with mortality outcomes. However, only four studies [10,11,12,13] provided survival-stratified IL-6 data in a format enabling calculation of standardized mean differences (means/medians with measures of dispersion for both survivor and non-survivor groups). The remaining four studies were excluded from meta-analysis for the following reasons: (1) Simpson 2000 endotoxin substudy [18] and LaRosa 2006 [19] analyzed overlapping patients from the Simpson 2000 main cohort [11], (2) Saikh 2020 [20] compared melioidosis patients to controls but did not stratify IL-6 by survival outcome, and (3) Wan 2025 [21] was a descriptive case series of three pediatric patients, all fatal, precluding comparison group analysis. The detailed study selection process is illustrated in the PRISMA flow diagram (Figure 1).

### 3.2. Characteristics of Included Studies

The 8 included studies were published between 1992 and 2025 and collectively enrolled a total of 920 patients with culture-confirmed melioidosis, including 351 non-survivors (overall mortality rate: 38.2%). The studies were predominantly prospective cohort studies (*n* = 9) conducted in hyperendemic regions, with the majority originating from Thailand (*n* = 6) and each from Sri Lanka, and China. Sample sizes ranged from 3 to 352 participants. IL-6 was measured at hospital admission (<24 h of presentation) in all studies, primarily from serum samples using immune assays which were ELISA, electrochemiluminescence (ECL) and B9 cell proliferation assay (Table 1).

Studies included in qualitative synthesis but excluded from meta-analysis fell into three categories: (1) Duplicate populations—Simpson et al., 2000 endotoxin substudy [18] used a subset of patients from the main Simpson 2000 cohort [11], and LaRosa 2006 [19] analyzed the same samples; including both would violate independence assumptions. (2) Insufficient outcome stratification—Saikh 2020 [20] measured IL-6 for diagnostic purposes (melioidosis vs. controls) but did not report survival-stratified IL-6 data. (3) Descriptive case series—Wan 2025 [21] reported three pediatric cases with extreme IL-6 elevations but lacked a comparison group, precluding effect size calculation.

Early work by Friedland et al. (1992) demonstrated that non-survivors had markedly elevated IL-6 at admission (median 4800 pg/mL) compared with survivors (<1000 pg/mL), and that levels above 1000 pg/mL predicted 75% mortality [10]. Larger cohorts from Thailand subsequently confirmed these findings: Simpson et al. (2000) reported that IL-6 was an independent predictor of death, with non-survivors showing significantly higher concentrations (*p* < 0.001) [11], while a companion sub-study of 68 patients provided clear stratification, with survivors having median IL-6 of 116 pg/mL versus 825 pg/mL in non-survivors [18]. LaRosa et al. (2006) further showed that IL-6 correlated with coagulopathy and a 40% mortality rate, although survival-specific IL-6 values were not reported [19]. More recent longitudinal data from Kaewarpai et al. (2020) demonstrated that persistently rising IL-6 trajectories predicted death, whereas survivors had declining levels over time [12], and Wright et al. (2021) validated IL-6 as an independent predictor of 28-day mortality across three cohorts, with improved predictive performance when combined with IL-8 (AUC up to 0.86) [13]. Complementary evidence also supports a diagnostic role: Saikh et al. (2020) reported excellent discriminatory ability of IL-6 for confirmed melioidosis versus controls (AUC 0.967), though survival was not assessed [20]. Other reports highlight extreme IL-6 elevation in special contexts, such as the pediatric cases with hemophagocytic lymphohistiocytosis described by Wan et al. (2025), all fatal with IL-6 levels exceeding 1400 pg/mL [21]. Taken together, these findings demonstrate that IL-6 is consistently elevated in melioidosis compared with controls and strongly associated with mortality, with both baseline levels and persistent elevations identifying patients at highest risk of death, thereby underscoring its potential as a prognostic biomarker and therapeutic target.

### 3.3. Quality of Included Studies

A total of eight studies were evaluated for methodological quality using the Newcastle–Ottawa Scale (NOS). Study quality varied across the included literature. One study (Kaewarpai 2020) was rated as low risk, reflecting its large prospective design, appropriate adjustment for confounders, and use of modern multiplex IL-6 assays [12]. Three studies (Wright 2021, LaRosa 2006, and Saikh 2020) were assessed as moderate risk, primarily due to smaller sample sizes, incomplete adjustment for confounders, or a diagnostic rather than prognostic focus [13,19,20]. Friedland (1992) and Simpson (2000) were judged to carry moderate-to-high risk of bias, owing to small sample sizes and reliance on older IL-6 bioassay or ELISA techniques [10,11]. Wan (2025), a descriptive case series, was included for quality assessment but not for quantitative synthesis [21]. The Simpson (2000, endotoxin sub-study) overlapped with its parent dataset and was therefore excluded to avoid duplication [18]. The details were in Supplementary File S2. In summary, four studies—Friedland (1992) [10], Kaewarpai (2020) [12], Simpson (2000) [11], and Wright (2021) [13]—were included in the quantitative meta-analysis.

### 3.4. Meta-Analysis of Circulating IL-6 and Mortality

**Figure 2:**
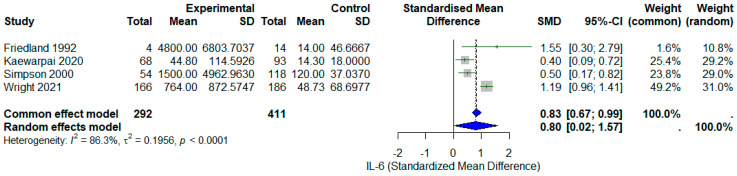
Forest plot of standardized mean differences (SMDs) in plasma IL-6 concentrations between non-survivors and survivors in melioidosis. Each study is represented by a square (proportional to study weight) with horizontal lines indicating 95% confidence intervals [10,11,12,13]. The diamond represents the pooled random effects estimate (SMD = 0.80, 95% CI 0.02–1.57). Positive SMD values indicate higher IL-6 levels in non-survivors. The vertical dashed line at SMD = 0 represents no difference between groups. Statistical significance (*p* < 0.05) is achieved when the confidence interval does not cross zero. Heterogeneity: *I*^2^ = 86.3%, τ^2^ = 0.1956, indicating substantial between-study variance. Gray square represents the SMD of each study. Blue diamond represents the pool SMD.

We performed a random effects meta-analysis (DerSimonian–Laird model with Hartung–Knapp adjustment) to estimate the standardized mean difference (SMD) in plasma IL-6 concentrations between non-survivors and survivors across eligible studies. The pooled SMD was 0.80 (95% CI: 0.02–1.57), indicating that IL-6 levels were significantly higher in non-survivors than in survivors (*I*^2^ = 86.3%, τ^2^ = 0.1956, *p* < 0.0001). As shown in Figure 2.

Given the different IL-6 measurement platforms, we examined whether effect estimates varied systematically by assay type. Studies using older methods (bioassay: Friedland 1992 [10], SMD = 1.55; ELISA: Simpson 2000 [11], SMD = 0.50) showed numerically larger effects than those using modern platforms (Luminex: Kaewarpai 2020 [12], SMD = 0.40; ECL: Wright 2021 [13], SMD = 1.19), though formal subgroup comparison was not statistically powered with only four studies. This pattern may reflect either (1) true secular trends in mortality rates or patient characteristics, (2) survival bias in older studies with higher mortality, or (3) differing dynamic ranges and sensitivity thresholds across platforms. Importantly, all four studies showed consistent direction of association (higher IL-6 in non-survivors), supporting a genuine biological phenomenon rather than assay artifact.

### 3.5. Leave-One-Out and Sensitivity Analysis

**Figure 3:**
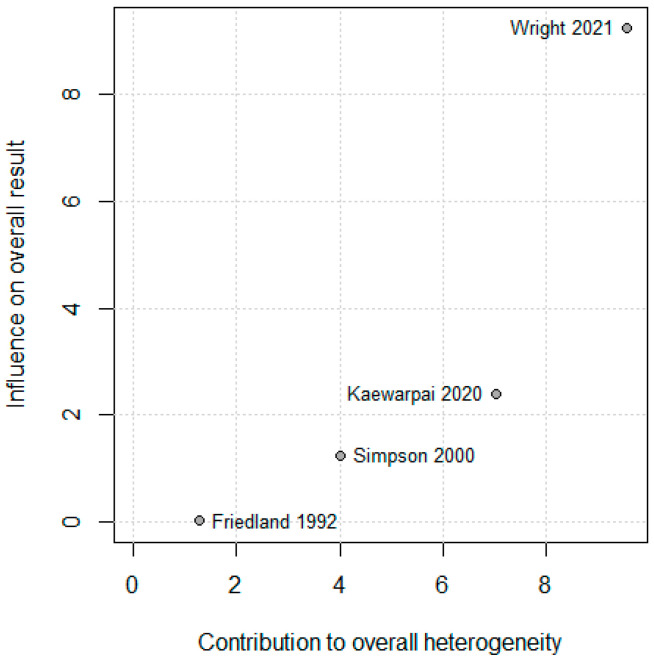
Baujat diagnostic plot assessing individual study contributions to heterogeneity and pooled effect estimate. The *x*-axis represents each study’s contribution to overall heterogeneity (Cochran’s Q statistic); the *y*-axis represents influence on the pooled SMD [10,11,12,13]. Studies positioned toward the upper right exert disproportionate influence on both heterogeneity and pooled estimate. Wright 2021 [13] contributes most to heterogeneity (x ≈ 9) due to its large sample size (*n* = 352) and methodological differences (electrochemiluminescence assay), making it an appropriate candidate for sensitivity analysis. This diagnostic plot helps identify outlier studies that may warrant separate consideration.

**Figure 4:**
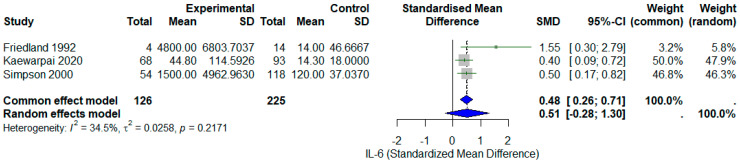
Sensitivity analysis forest plot excluding Wright et al., 2021 [13]. Removal of the largest study (*n* = 352) reduces the pooled SMD from 0.80 to 0.51 and heterogeneity from *I*^2^ = 86.3% to 34.5%, demonstrating that methodological differences in this study (electrochemiluminescence platform, multicohort validation design) contributed substantially to between-study variance [10,11,12]. However, the direction of association remains consistent (higher IL-6 in non-survivors), supporting the robustness of the overall finding despite loss of statistical significance due to reduced sample size. Gray square represents the SMD of each study. Blue diamond represents the pool SMD.

The leave-one-out sensitivity analysis showed that exclusion of any single study did not materially alter the pooled standardized mean difference (SMD), confirming the robustness of the overall meta-analytic estimate. Wright (2021) [13] contributed modestly more to heterogeneity (Baujat x ≈ 9), as shown in Figure 3. Because Wright (2021) [13] contributed most to heterogeneity in the Baujat plot (Baujat x ≈ 9; Figure 4), a sensitivity analysis was conducted after excluding this study. The pooled standardized mean difference (SMD) decreased from 0.80 (95% CI 0.02–−1.57) to 0.51 (95% CI −0.28–−1.30), while heterogeneity dropped markedly from 86.3% to 34.5%, as shown in Figure 4. Although statistical significance was lost after exclusion, the direction of the association remained consistent—higher IL-6 levels in non-survivors.

This sensitivity analysis provides several important insights for clinical interpretation. First, the substantial reduction in heterogeneity (from 86.3% to 34.5%) when excluding Wright 2021 [13] suggests that methodological differences—particularly the use of electrochemiluminescence assay in Wright versus ELISA/bioassay in earlier studies—contribute meaningfully to between-study variance. Second, the loss of statistical significance reflects reduced statistical power rather than reversal of effect direction, as the point estimate remained positive (SMD = 0.51). Third, the consistency of effect direction across all four studies, regardless of assay platform, clinical setting, or study period (1992–2021), supports a genuine biological association between IL-6 elevation and mortality. Clinically, these findings suggest that IL-6’s prognostic value is generalizable but that establishment of specific diagnostic thresholds will require assay-specific calibration and validation.

### 3.6. Assessment of Publication Bias and Meta-Regression

Formal assessment of publication bias using funnel plots and Egger’s regression test, as well as meta-regression analyses, was not conducted because fewer than ten studies were available for quantitative synthesis. According to established methodological guidance, these approaches lack sufficient power and reliability when applied to small numbers of studies and may yield misleading results [22,23].

## 4. Discussion

This systematic review and meta-analysis demonstrate that circulating interleukin-6 (IL-6) is a consistent and biologically plausible prognostic biomarker for mortality in melioidosis. Across four studies comprising 411 patients, IL-6 concentrations were significantly higher in non-survivors compared with survivors, with a pooled standardized mean difference (SMD) of 0.80 (95% CI 0.02–1.57; *I*^2^ = 86.3%). This finding underscores IL-6’s central role in the hyperinflammatory response characteristic of severe *Burkholderia pseudomallei* infection. As with other sepsis syndromes, excessive host immune activation, rather than bacterial burden alone, appears to be the major determinant of fatal outcomes in melioidosis [11,13].

Clinicians currently rely on several readily available laboratory markers to assess disease severity and prognosis in sepsis, including white blood cell count, C-reactive protein (CRP), procalcitonin, and lactate. In the context of melioidosis specifically, Wright et al. (2021) demonstrated that IL-6 combined with IL-8 provided superior prognostic discrimination (AUC > 0.85) compared with clinical severity scores alone [13]. While CRP and procalcitonin are widely used in sepsis management, they reflect hepatic acute-phase responses and may lag behind the immediate cytokine storm that characterizes early severe disease. IL-6, as a proximal inflammatory mediator directly released from activated monocytes and macrophages in response to pathogen recognition, may provide earlier and more mechanistically specific information about dysregulated host responses.

However, practical implementation of IL-6 testing faces challenges. Unlike CRP or complete blood counts, IL-6 assays are not routinely available in most clinical laboratories, particularly in resource-limited endemic regions where melioidosis burden is highest. Point-of-care IL-6 testing platforms are emerging but not yet validated for melioidosis prognostication. Furthermore, IL-6 exhibits substantial diurnal variation and is influenced by multiple non-infectious conditions (e.g., trauma, malignancy, autoimmune disease), potentially limiting specificity in undifferentiated critically ill patients. The optimal clinical application may therefore be as part of multiplex cytokine panels that capture complementary aspects of immune dysregulation, rather than as a standalone test [11]. These findings are consistent with broader sepsis literature emphasizing IL-6 as a key mediator and biomarker of inflammatory severity [8].

Beyond single time point measurements, dynamic IL-6 trajectories also have prognostic significance. Kaewarpai et al. (2020) showed that non-survivors displayed persistently rising IL-6 levels, whereas survivors had declining cytokine concentrations over time [12]. This temporal pattern suggests that failure to suppress IL-6 reflects sustained immune dysregulation and impaired resolution of inflammation. Comparable observations in community-acquired pneumonia and general sepsis cohorts reinforce the importance of cytokine kinetics in mortality prediction [24].

Integration of IL-6 into multiplex biomarker panels may further enhance predictive accuracy. Wright et al. (2021) demonstrated that combining IL-6 and IL-8 improved discrimination of 28-day mortality compared with IL-6 alone [13]. The complementary roles of IL-6 and IL-8—systemic inflammation and neutrophil chemotaxis, respectively—offer a mechanistic rationale for this improvement [14,16]. Thus, IL-6 not only serves as a robust individual marker but also as a foundational element of multi-analyte prognostic models for melioidosis.

The substantial heterogeneity observed in our meta-analysis (*I*^2^ = 86.3%) likely reflects multiple interconnected methodological and clinical factors. Assay methodology represents a major source of variability, as the four included studies employed fundamentally different IL-6 quantification platforms with distinct performance characteristics. Friedland et al. (1992) used the B9 cell proliferation bioassay with sensitivity of approximately 1 pg/mL [10], Simpson et al. (2000) employed enzyme-linked immunosorbent assay (ELISA) with detection limits of 5–10 pg/mL [11], Kaewarpai et al. (2020) utilized modern bead-based Luminex multiplex technology [12], and Wright et al. (2021) applied electrochemiluminescence immunoassay [13]. Each platform uses different calibration standards, antibody specificities, and dynamic ranges, which can produce systematic variations in absolute IL-6 concentrations even when measuring identical samples. Patient characteristics also varied considerably across studies, with diabetes mellitus prevalence ranging from 30% to 74% and overall mortality rates spanning 22% to 55%, indicating substantial differences in baseline disease severity and metabolic comorbidity profiles. Sample timing, while standardized to hospital admission in all studies, exhibited variable precision regarding the actual time from symptom onset, with some studies collecting blood within hours of presentation and others potentially capturing patients up to 24–48 h after initial hospitalization. Finally, clinical severity indicators differed substantially, including variations in APACHE II scores, bacteremia rates, presence of septic shock, and patterns of organ failure, all of which influence both cytokine responses and mortality risk. Collectively, these methodological and clinical sources of heterogeneity help explain the substantial between-study variance observed in our pooled analysis, though the consistent direction of association across all studies—higher IL-6 in non-survivors—supports a genuine biological phenomenon underlying the statistical heterogeneity.

The sensitivity analysis excluding Wright (2021) [13] reduced heterogeneity to 34.5% and produced a lower pooled SMD (0.51 [95% CI: 0.28–1.30]), yet the direction of association remained consistent. This confirms that although Wright’s large dataset influenced the magnitude and precision of the pooled effect, the overall conclusion—higher IL-6 levels in non-survivors—remains robust.

Extremely elevated IL-6 concentrations have also been observed in specific clinical contexts, such as pediatric hemophagocytic lymphohistiocytosis secondary to melioidosis (IL-6 > 1400 pg/mL) [21]. These extreme elevations likely represent cytokine storm syndromes, highlighting IL-6’s pathogenic as well as prognostic importance.

Experimental data further support IL-6’s mechanistic role in the host response to *B. pseudomallei*. In vitro stimulation of peripheral blood mononuclear cells from acute myeloid leukemia patients induced substantial IL-6 release [25], and murine models have demonstrated that IL-6 mRNA expression parallels disease severity [26]. Together, these findings emphasize IL-6 as a central mediator of host–pathogen interaction and immune dysregulation in melioidosis.

Translation of IL-6 measurement into routine clinical practice for melioidosis management presents both opportunities and challenges that warrant detailed consideration. Timing of measurement is critical: our analysis focused on admission samples, which provide the earliest prognostic information when clinical decision-making is most consequential. Serial IL-6 measurements may offer additional value, as Kaewarpai et al. demonstrated that rising trajectories predict mortality whereas declining levels indicate recovery [12]. However, this requires repeated phlebotomy and laboratory resources that may be limited in endemic regions.

Role in clinical triage and risk stratification represents the most immediate application. In resource-constrained settings where ICU beds are scarce, IL-6 levels could help identify patients requiring intensive monitoring or early transfer to tertiary centers. Wright et al. showed that combining IL-6 with IL-8 improved risk prediction beyond clinical scores alone (AUC 0.86 vs. 0.78) [13], suggesting that multiplex cytokine panels could enhance existing severity assessment tools like APACHE II or SOFA scores. Integration into clinical algorithms might follow a tiered approach: first-line triage using readily available markers (lactate, CRP, clinical scores), followed by IL-6/IL-8 measurement in intermediate-risk patients where additional prognostic information would meaningfully alter management.

Guiding immunomodulatory therapy represents a more speculative but potentially transformative application. If dysregulated IL-6 contributes mechanistically to mortality (not merely serving as a marker), then targeted IL-6 blockade with agents such as tocilizumab—already approved for cytokine release syndrome in CAR-T therapy—might improve outcomes in severe melioidosis. However, premature immunosuppression could also impair bacterial clearance. IL-6 thresholds could potentially identify patients with maladaptive hyperinflammation most likely to benefit from adjunctive immunomodulation, though this hypothesis requires validation in randomized controlled trials.

Practical implementation barriers include limited IL-6 assay availability in endemic regions (Thailand, Northern Australia, South Asia), lack of standardized platforms and reference ranges, cost considerations, and need for cold chain and specialized equipment. Point-of-care IL-6 testing platforms are emerging but require validation specifically in melioidosis populations. Realistically, near-term implementation is most feasible in tertiary referral centers managing severe cases, with potential expansion as technology and infrastructure develop.

Several important limitations must be acknowledged. First, the small number of eligible studies (*n* = 4) for quantitative synthesis limits statistical power for subgroup analyses and precludes formal assessment of publication bias using funnel plot asymmetry or Egger’s regression test [22,23]. While eight studies were identified in qualitative review, only four provided survival-stratified IL-6 data suitable for meta-analysis. This reflects the broader challenge that many melioidosis cohort studies measure multiple cytokines but do not consistently report survival-stratified concentrations for each analyte. Second, reliance on single time point measurements represents a significant conceptual limitation. IL-6 exhibits marked temporal dynamics during sepsis, with levels typically peaking within 24 h of infection onset and declining with effective treatment or progressing to persistently elevated levels in patients developing multi-organ failure [12]. Our meta-analysis captured only admission values, missing potentially important prognostic information from cytokine trajectories. Kaewarpai et al. demonstrated that non-survivors exhibited rising IL-6 over days 0–5–12 whereas survivors showed declining levels [12], suggesting that kinetic patterns may be more informative than absolute baseline concentrations. Future studies should employ serial sampling with joint modeling or trajectory analysis approaches to capture temporal evolution of inflammatory responses. Point-of-care platforms enabling repeated bedside IL-6 measurement could make such monitoring clinically feasible. Third, potential unmeasured confounding may have influenced observed associations. Antibiotic administration timing relative to blood sampling was not consistently reported; early antibiotic therapy can alter cytokine release patterns, particularly for cell wall-active agents in Gram-negative infections. Diabetes mellitus, present in 30–74% of melioidosis patients, fundamentally alters both baseline cytokine profiles and dynamic inflammatory responses to infection [14]. Yet only Wright et al. explicitly adjusted for diabetes in multivariable models [13]. Co-infections (particularly with other endemic pathogens like leptospirosis or dengue in tropical settings) could independently elevate IL-6, though all included studies required culture-confirmed *B. pseudomallei*. Source of infection (bacteremic vs. localized) and anatomic site (pneumonia vs. abscess) likely influence systemic cytokine levels but were not uniformly available for stratified analysis. Immunosuppression from conditions like malignancy, HIV, or chronic kidney disease may blunt IL-6 responses, creating survival bias if immunocompromised patients died before developing high IL-6 levels. Fourth, geographic limitations are substantial. Six of eight studies originated from Thailand, with single studies from Sri Lanka and China. Generalizability to other endemic regions (Northern Australia, South Asia, Brazil) or emerging areas (temperate regions experiencing climate change) remains uncertain. Differences in predominant *B. pseudomallei* sequence types, antibiotic resistance patterns, healthcare infrastructure, and population genetics may all modify IL-6’s prognostic performance.

The inability to formally assess publication bias due to insufficient study numbers (*n* = 4) represents an important limitation. Studies demonstrating statistically significant prognostic associations may be more likely to report survival-stratified cytokine data, potentially creating selection bias even within our restrictive inclusion criteria. The field of melioidosis research may be particularly susceptible to this bias, as large cohort studies often measure multiple cytokines but selectively report only those showing significant associations with outcomes. However, the inclusion of studies spanning 33 years (1992–2025) with consistent findings despite evolving publication standards provides some reassurance against temporal publication bias. Prospective registration of future biomarker studies with pre-specified analysis plans would mitigate this concern.

Despite these limitations, the convergence of evidence across decades and diverse methodologies strongly supports IL-6 as a meaningful prognostic biomarker in melioidosis. Its integration into clinical workflows—particularly as part of multiplex cytokine panels—could enhance early risk stratification, inform targeted monitoring, and guide immunomodulatory therapies in severe disease.

## 5. Conclusions

This meta-analysis of four studies (411 patients) demonstrates that elevated interleukin-6 (IL-6) levels are consistently associated with mortality in melioidosis. Although heterogeneity was substantial (*I*^2^ = 86.3%), the association remained directionally consistent across all studies, with sensitivity analysis reducing heterogeneity to 34.5%. IL-6 represents a promising prognostic biomarker reflecting the hyperinflammatory response in severe *Burkholderia pseudomallei* infection. However, the limited number of studies necessitates cautious interpretation.

Future research priorities include: (1) prospective multicenter validation studies with standardized assay protocols to establish prognostic thresholds across endemic regions, (2) longitudinal studies with serial measurements to determine whether IL-6 trajectories outperform single time point assessments, (3) comparative effectiveness trials evaluating whether IL-6-guided risk stratification improves clinical outcomes, and (4) randomized controlled trials of IL-6-targeted immunomodulation (e.g., tocilizumab) in patients with marked elevation. Integration of IL-6 into clinical guidelines for melioidosis management awaits completion of these validation studies but represents a promising precision medicine approach for this neglected tropical disease.

## Figures and Tables

**Table 1:** Characteristics of Included Studies.

Author, Year [Ref]	Country	Study Design	Sample Size	Population Details	IL6 Measurement Method	IL6 Levels Reported	Outcome Measures	Mortality Rate	Association IL6 Outcome	Effect Estimates	Notes on Bias or Confounders	Include in Meta-Analysis
Friedland et al., 1992 [10]	Thailand	Prospective observational cytokine study	18	Patients with septicemic (*n* = 8) and localized (*n* = 8) *Pseudomonas pseudomallei* infection; 2 E. coli septicemia for comparison.	Plasma IL-6 measured by B9 cell proliferation assay (sensitivity 1 pg/mL); serial samples up to 30 days.	Median admission IL-6: non-survivors 4800 pg/mL (range 60–9245), survivors much lower (*p* = 0.007). Elevated IL-6 (>1000 pg/mL) predicted 75% mortality.	Mortality prediction; longitudinal IL-6 and IL-8 dynamics	22% (4/18 overall; 4/10 septicemic died, all within 24 h)	High IL-6 at admission strongly predictive of mortality. IL-6 remained persistently elevated in all patients during hospitalization.	Threshold effect: IL-6 > 1000 pg/mL associated with 75% mortality (*p* = 0.011).	Small sample size; older cytokine assays; single hospital; some patients died before serial samples could be obtained.	Yes
Kaewarpai et al., 2020 [12]	Thailand	Prospective longitudinal cohort	161	Adults with culture-confirmed melioidosis (81% bacteremic, 74% diabetic). Controls: 13 healthy, 11 diabetics.	Plasma cytokines (IL-6, IL-8, IL-10, IFN-γ, TNF-α, IL-17A, IL-23, etc.) measured by bead-based multiplex (Luminex) at days 0, 5, 12, 28.	Median IL-6 higher in non-survivors at day 0 (*p* < 0.001). Non-survivors had rising IL-6 over time, survivors declining. AUROC IL-6 = 0.75 (95% CI 0.67–0.82).	28-day mortality prediction, longitudinal IL-6 dynamics	42% (68/161)	IL-6 significantly higher in non-survivors vs. survivors. Persistent/increasing IL-6 trajectory predicted death (joint modeling *p* < 0.004).	Effect estimates: AUROC IL-6 = 0.75 (95% CI 0.67–0.82). Independent temporal predictor of survival.	Single-country, hospitalized adults; cytokine sampling only after culture confirmation (delayed in some); no external validation.	Yes
LaRosa et al., 2006 [19]	Thailand	Prospective biomarker analysis (subset of RCT)	30	Acute severe melioidosis, 40% diabetic, 47% bacteremia, 30% lung involvement	Plasma IL-6 (ELISA, previously published dataset)	Median 240.2 pg/mL (range 14.6–745,000; IQR 64.9–1646.5)	Mortality, shock, renal failure, liver dysfunction	40% (12/30)	Higher IL-6 inversely correlated with protein C (ρ = –0.74) and antithrombin (ρ = –0.68); prognostic value for poor outcomes	Not directly reported for IL-6 vs. mortality; IL-6 correlated with severity biomarkers	Subset analysis, small sample size, possible selection bias	No (Same samples with Simson et al., 2000) [11]
Saikh et al., 2020 [20]	Sri Lanka	Observational biomarker study	116	Confirmed melioidosis patients (*B. pseudomallei* culture positive), with additional probable, relapsed, and convalescent cases included for comparison.	Serum cytokines measured by Meso Scale Discovery (MSD) ultrasensitive assay; IL-6 levels compared across groups	IL-6 markedly elevated in confirmed and probable melioidosis: IQR ~2.66–197.62 pg/mL (confirmed) vs. 0.30–0.50 pg/mL (controls). ROC AUC for IL-6 = 0.967, cut-off 0.70 pg/mL.	Diagnosis of melioidosis vs. controls; differentiation from other infections (sepsis, leptospirosis)	Not directly reported	IL-6 consistently higher in melioidosis cases; strong discriminatory power with AUC 0.967; better than TNF-α, IL-1β, IFN-γ.	Effect estimates: AUC 0.967, sensitivity/specificity high at diagnostic cut-off (Youden index 0.849).	Cross-sectional, limited to Sri Lankan cohort, potential confounders from co-infections; IL-6 not tested as mortality predictor in this study.	No (diagnostic biomarker study; no mortality outcomes)
Simpson et al., 2000 [11]	Thailand	Prospective cohort (subset of RCT comparing imipenem vs. ceftazidime)	172	Adult Thai patients with severe melioidosis, admitted to Sappasitprasong Hospital (1994–1997).	Plasma IL-6 measured by ELISA (detection limit 5–10 pg/mL)	Median baseline IL-6: 227.2 pg/mL (range <5–745,000; IQR 82.6–708.2). Higher in non-survivors vs. survivors (*p* < 0.001).	Mortality, septicemia, APACHE II score, plasma lactate	31.4% (54/172); 48% mortality in bacteremic subgroup	IL-6 strongly associated with mortality (OR 3.60 per log increase; 95% CI 1.76–7.38, *p* < 0.001). Independent predictor of outcome alongside APACHE II score.	Multivariate: APACHE II (OR 1.17 per point) + IL-6 remained predictors; IL-10 lost significance when both included. IL-6/IL-10 ratio associated with outcome in univariate but not independent.	Large cohort, single hospital; cytokine levels measured at baseline only; antibiotic treatment arms balanced.	Yes
Simpson et al., 2000 (Endotoxin study) [18]	Thailand	Randomized trial substudy (imipenem vs. ceftazidime)	68	Adults with severe septicemic melioidosis randomized to ceftazidime or imipenem.	Baseline and post-antibiotic cytokines (IL-6, TNF-α, IL-10) measured by ELISA; endotoxin measured by turbidimetric LAL assay.	Median baseline IL-6: Survivors 116.5 pg/mL (IQR 43–245); Non-survivors 825 pg/mL (IQR 229–2829), *p* < 0.001.	Mortality during hospitalization; endotoxin and cytokine release after antibiotics	35% (24/68)	IL-6 strongly predictive of death (baseline higher in non-survivors, *p* < 0.001). IL-6 correlated with TNF-α and IL-10, but not endotoxin levels.	Effect estimate: IL-6 median non-survivors 825 vs. survivors 116 pg/mL (*p* < 0.001). No difference by antibiotic group.	Small sample; cytokines measured only up to 6 h post-antibiotics; single hospital study.	No (Sub study of Simson 2000) [11]
Wan et al., 2025 [21]	China (Hainan Island)	Case series (3 pediatric cases in one family)	3	Three children (ages 4, 6, 12) with culture-confirmed B. pseudomallei septicemia complicated by HLH.	Serum cytokines (IL-2, IL-4, IL-6, IL-10, IL-17A, IFN-γ, TNF-α) measured; blood culture confirmed B. pseudomallei.	IL-6 extremely elevated: 1453–2031 pg/mL in two cases; IL-10 > 5700 pg/mL in one case; all had cytokine storm profile.	Mortality, HLH complications, multi-organ failure	100% (3/3)	Markedly high IL-6 and IL-10 levels during cytokine storm; associated with HLH and fatal outcome.	Not quantified as OR; descriptive case series with biomarker table.	Small case series, familial cluster, no controls; findings not generalizable but highlight IL-6 role in cytokine storm with HLH.	No (case series, descriptive only)
Wright et al., 2021 [13]	Thailand	Prospective biomarker cohort (derivation, validation, external validation)	352	Hospitalized adults with culture-confirmed melioidosis; derivation set (113), internal validation (78), external validation (161).	Plasma cytokines (IL-6, IL-8, IL-10, TNF-α, IFN-γ, G-CSF, IL-17A, IL-1β) measured by electrochemiluminescence and bead-based multiplex assays.	Median IL-6 (derivation): Survivors 41 pg/mL (IQR 16–239), non-survivors 1938 pg/mL (IQR 132–1980). Validation: Survivors 44 pg/mL, non-survivors 948 pg/mL. External: Survivors 14 pg/mL, non-survivors 45 pg/mL (*p* < 0.001 across cohorts).	28-day mortality prediction	Overall mortality ~42–55% across cohorts	IL-6 independent predictor of 28-day mortality (OR 3.62; 95% CI 1.97–6.66; *p* < 0.001). IL-6 + IL-8 biomarker model improved AUC from 0.78 to 0.86 (derivation), validated in external cohorts (AUC 0.81).	IL-6 significantly associated with mortality even after adjusting for comorbidities and SOFA score. IL-6 + IL-8 performed comparably to clinical risk models.	Robust multi-cohort validation; single-time point cytokine measurement; assay differences across cohorts.	Yes (Separate to 3 studies; derivation, validation, and external validation)

Abbreviations: IL, interleukin; ELISA, enzyme-linked immunosorbent assay; RCT, randomized controlled trial; TLR, toll like receptor; CI, confidential interval, TNF, tumor necrosis factor; IQR, interquartile range; OR, odd ratio.

## Data Availability

No new data were created.

## Supplementary Materials

The following supporting information can be downloaded at: https://www.mdpi.com/article/10.3390/diseases13120385/s1, Supplementary File S1: Search strategies; Supplementary File S2: Quality assessment (NOS).

## Abbreviations

The following abbreviations are used in this manuscript:

IL-6	Interleukin 6
ELISA	Enzyme-linked immunosorbent assay
ELC	Electrochemiluminescence
SMD	Standardized mean differences
ROM	Ratio of means
